# Psychosocial Stress Profiles and Cognitive Function in a Racially and Ethnically Diverse Sample of Adults

**DOI:** 10.1093/geroni/igab046.292

**Published:** 2021-12-17

**Authors:** Elizabeth Munoz, Laura Zahodne, Richard Gonzalez, Noah Webster, Martin Sliwinski, Stacey Scott

**Affiliations:** 1 The University of Texas at Austin, The University of Texas at Austin, Texas, United States; 2 University of Michigan, Ann Arbor, Michigan, United States; 3 The Pennsylvania State University, University Park, Pennsylvania, United States; 4 Stony Brook University, Stony Brook, New York, United States

## Abstract

We developed comprehensive multi-domain profiles of psychosocial stress in urban-dwelling, racially and ethnically diverse adults (age range: 25-65; N=256; 63% Non-Hispanic Black; 25% Hispanic; 9% Non-Hispanic White) and evaluated associations with cognitive function. Participants completed psychosocial stress measures tapping into ten domains and tasks of processing speed, working memory, and episodic memory. Latent profile analyses controlling for age yielded four-profiles: high neighborhood stress, moderate versus high work stress and daily discrimination, and high health and relationship stress. Profiles significantly differed in income, age, and employment status. The profile with moderate work stress and daily discrimination and the profile with high neighborhood stress each had significantly lower working memory than the other profiles. The finding of lower working memory among individuals in the moderate work stress and daily discrimination profile was not due to sociodemographic variables. Results highlight the potentially cumulative influence of different contextual stressors on cognition.

